# Honor the dice: narrative effects in “actual-play” media

**DOI:** 10.3389/fpsyg.2026.1780225

**Published:** 2026-04-01

**Authors:** Emma J. Bognar, Kristi A. Costabile

**Affiliations:** Social Cognition and Perception Laboratory, Department of Psychology, Iowa State University, Ames, IA, United States

**Keywords:** narrative effects, parasocial experiences, role-playing games, self-determination theory, transportation

## Abstract

Tabletop role-playing games allow players to create improvised, collaborative narratives using codified game systems. “Actual-play” shows feature professional actors utilizing these game systems to create long-form narratives. The present research sought to examine traditional narrative engagement processes, basic need satisfaction, and motivation to continue viewing among first-time viewers of this emerging genre of entertainment media. First-time viewers were randomly assigned to attend to narrative features of the show (e.g., plot) or to game mechanics. Results indicated that viewers assigned to focus on narrative features experienced greater transportation, parasocial interaction, and relatedness with characters than did those assigned to focus on game mechanics. Narrative engagement processes were in turn associated with greater interest in viewing additional show episodes. This work highlights the importance of storytelling in collaborative games. Findings have implications for enhancing viewer enjoyment as well as reducing barriers to enjoying emerging media.

## 1 Introduction

From cave paintings and oral stories, to illuminated manuscripts and printed books, to radio plays and film, to modern forms of interactive narratives (e.g., video games), entertainment narratives have been found to fulfill audience members’ social and personal goals (Dubourg and Baumard, 2022; Vorderer and Halfmann, 2019). Analog role-playing games have grown in popularity over the last few decades, perhaps acting as a modern successor to ancestral folk narratives (Cragoe, 2016). Most recently, role-playing games have transformed from self-directed entertainment to other-directed entertainment in which audiences are invited to observe actors playing the game. We aimed to explore how narrative processes (e.g., transportation, parasocial interaction, and need satisfaction) operate for audiences of this new form of collaborative, improvisational media. Given the barriers to engaging with a new, complex form of entertainment (Rogers, 2003), we sought to examine how immersive qualities of such narratives may facilitate viewer engagement.

Actual-play shows consist of professional entertainers using table-top roleplaying games to tell stories for audience entertainment. These series have been credited for increasing engagement with table-top roleplaying games in recent years (Chalk, 2022; Cruz, 2024), and shows are being adapted into mainstream media (i.e., animated series; Whitten, 2019). Due to its rise in popularity, we examined the entertainment experiences of viewers of actual-play shows and the characteristics of the games that might attract new viewers to the medium.

Table-top roleplaying games (TTRPGs) can be described as a gamified system for improvised, collaborative storytelling (Deterding and Zagal, 2018). Games often include a person who acts as a Game Master who creates the overarching narrative and enforces game rules. In many game systems, success or failure of a given action is determined by a dice roll and a character-specific modifier (e.g., a character skilled in deception might add +7 to their dice roll when trying to lie to someone). Tabletop role-playing games are evolving, transforming from interactive and self-directed media to media performed for others. Thus, the *actual-play* show invites not only players, but also audience members to experience the collaborative narrative as it is built.

Although the detailed plots and developed characters may closely resemble traditional entertainment narratives (e.g., film), actual-play shows involve long-form improvisational storytelling. Additionally, actual-play shows depict individuals playing a game with its own set of rules and the narrative space is created almost entirely via oration, wherein players typically remain seated around a single table for the duration of the show. Accordingly, the show might lack immersive qualities as the visual presentation is a constant reminder of the pretensive world. Moreover, actual-play shows create several layers of simultaneous communication. For example, a player may be speaking as themselves and discuss the use of game mechanics or the player might communicate “in-character” in which they speak as their character and describe their actions (Decicio, 2020). As such, actual-play shows occupy a unique space in the entertainment industry, combining elements from beloved improvisational shows with ancient practices such as oral storytelling (Cragoe, 2016).

Narrative audiences easily immerse themselves in stories, forming connections with narrative characters and experiencing genuine emotions in response to the depicted events (Djikic et al., 2009; Green and Brock, 2000; Tukachinsky et al., 2020). Psychological transportation occurs when a narrative audience member has psychologically entered the story world. The audience member’s attention is fully attuned to the story and awareness of their physical reality dims (Green and Brock, 2000). When transported, the viewer^1^ responds cognitively and emotionally to elements present in the story world. Transportation is a foundational narrative process, occurring across narrative media modalities and contributing to enjoyment (Green et al., 2008).

Parasocial interactions can be conceptualized as an early stage in the development of more advanced parasocial engagement (e.g., parasocial relationships, Tukachinsky and Stever, 2019). We conceptualize parasocial interactions as one-sided interactions in which an audience member responds to a narrative character (Liebers and Schramm, 2019), such as when an audience member tells a character in a horror movie not to go into a dark room (e.g., “Don’t go in there!”). Parasocial relationships confer a variety of benefits for the audience members, (Gardner and Knowles, 2008) such as buffering against effects of rejection (Derrick et al., 2009) and facilitating development toward an ideal self (Derrick et al., 2008).

Media narratives may be particularly appealing because they satisfy the audience’s basic psychological needs (Costabile et al., 2018). Self-determination theory proposes three innate needs fundamental to motivation: autonomy (i.e., self-direction), relatedness (i.e., social connection), and competence (i.e., sufficient ability; Deci and Ryan, 2000). Satisfaction of these basic needs has been proposed as an explanation for motivation to engage with media narratives (Adachi et al., 2018; Oliver et al., 2016; Ryan et al., 2006).

Satisfaction of relatedness needs might be especially relevant for viewers of actual-play narratives given the multilayered experiences of parasocial interactions. The many layers of communication allow the audience to engage not only with the characters of the game, but also to feel included in the act of play (Decicio, 2020). The satisfaction of competence needs via viewing actual-play shows might occur not only through the comprehension of particularly complex narratives, but also through a mastery of the game mechanics. To fully understand an actual-play show, the viewer must follow the plot as well as learn the rules of the game. In this way, actual-play shows may offer unique opportunities for competence need satisfaction that are unavailable in traditional narrative media. Of course, we must acknowledge that competence need satisfaction, as studied in the self-determination theory literature, typically relies on competencies in interactive or active tasks (e.g., work settings or video games; Deci and Ryan, 2000). In the present case, the competency is more likened to observational competence, which Norris (1984) described as including the competence to *make observations well*. There has been less work investigating competence need satisfaction in this area than relatedness need satisfaction, however some researchers have explored the potential of “viewing competence” in audiences (Adachi et al., 2018). Viewers may experience need satisfaction from making complex observations and understanding them.

Despite the improvisational narrative and the impoverished physical environment, actual-play shows offer audiences engaging stories in which to be transported, actors and characters with which to relate, and opportunities to satisfy the audience’s competence and relatedness needs through story comprehension and engagement with actors/characters.

By their improvisational nature and layered with gameplay mechanics, actual-play shows are complex. Complexity is generally understood to have a negative impact on the adoption process for new technology and media (Rogers, 2003). The present investigation aimed to mitigate the effects of this complexity by directing first-time viewers’ attention to narrative features, a more familiar and accessible aspect of the show. By focusing on first-time viewers, effects of familiarity with the show or game conventions will be reduced. Understanding this early stage of engagement with the novel media is a critical period of inquiry to attract new audiences. Focus on narrative features might also reduce the potential distraction of narrative engagement when attempting to learn game mechanics. As such, we hypothesized the following: participants instructed to attend to the narrative would report experiencing more transportation than those instructed to attend to the game mechanics. We also predicted that parasocial interaction would be reported as higher for those focusing on the narrative. Relatedness need satisfaction was expected to be higher for those focused on the narrative, and relatedness need satisfaction was predicted to be positively associated with parasocial interaction. Additionally, we expected competence need satisfaction to be positively associated with (a) mechanics comprehension and (b) plot comprehension. Finally, we hypothesized that those instructed to focus on the narrative would demonstrate higher comprehension of the plot than those instructed to focus on the mechanics.

## 2 Methods

Study design, hypotheses, and analysis plans were preregistered (https://osf.io/v2xj7/?view_only=7d72027c9aa04377b9611cae4b868314). Supplementary materials are available on OSF (https://osf.io/4px9w/?view_only=974691959751464b80ebd7b4e0852727).

### 2.1 Design

Participants were randomly assigned to one of two attention focus conditions: narrative plot-focus condition or mechanics-focus condition. Those in the plot-focus condition were instructed to attend to the plot, while those in the mechanics-focus condition were given a behavioral task to direct their attention toward the gameplay elements of the show. Further details are available below in the Procedure section.

### 2.2 Participants

A power analysis using G*Power version 3.1.9.6 (Faul, 2020; Faul et al., 2007) at 0.80 power for a two-tailed analysis assuming an effect size of *d* = 0.40 with a standard alpha error probability of 0.05 recommended a sample size of *N* = 200. We recruited 235 undergraduates from a large university in the midwestern United States who received course credit for participation. In accordance with our preregistration plan, data from those who failed attention checks, were outliers on 2 or more scales, who failed to adequately engage with the behavioral task, or were actual-play experts^2^ were removed from analyses leaving a sample of 174, see full details in supplemental materials. A post-hoc power analysis (Faul et al., 2007) indicated that the final sample size could detect differences of *d* = 0.40 with a power of 0.74. This is a reduction of 6% to detect the *a priori* effect size of interest (80 to 74%), see supplementary analyses using the full sample on OSF.

The final sample of 174 participants ranged in age from 18 to 22 years old (*M* = 19.17, *SD* = 0.95), and included 112 women, 61 men, and 1 participant who identified as non-binary. The majority of the sample identified as white (*N* = 140).

### 2.3 Procedures

All materials were presented and completed on Qualtrics. All procedures were completed within 60 min.

Following informed consent, participants were provided with a description of actual-play shows and a summary of the episode plot. They then received instructions regarding their experimental condition and watched an abridged version of the episode. Participants completed measures of psychological transportation, parasocial interaction, basic need satisfaction, and intrinsic viewing motivation (presented in this order). These measures included two attention check items (e.g., “We want to ensure people are reading carefully, please select Agree for this item”). Then, participants answered questions assessing their comprehension of the narrative plot and game mechanics. Then, participants answered questions assessing comprehension of narrative plot and game mechanics. Finally, the participants completed measures assessing experience with role-playing games, demographics, and whether they would like to receive a link to the original version of the stimulus. Full materials are provided on OSF.

#### 2.3.1 Experimental manipulation

Participants in the plot-focus condition were instructed to focus on the narrative of the episode, including character relationships and goals, and the overarching plot.

To ensure attention was directed toward mechanics, participants in the mechanics-focus condition completed a behavioral task while watching the stimulus. In a computer window adjacent to the video stimulus, participants were instructed to record a list of the dice rolls that occurred during the episode, both the reason for the roll (e.g., “Perception check”) and the total score (e.g., “17”), see supplemental materials on OSF for an example.

### 2.4 Materials

#### 2.4.1 Stimulus

The stimulus used in this study was an edited version of a popular actual-play show (Dimension 20: *The Unsleeping City*; Dropout, 2019). The first episode of the season was edited to a length of 27 min and 42 s, and included character introductions, role-playing, and gameplay mechanics. There were 28 discernable dice rolls in this edit.

#### 2.4.2 Measures

##### 2.4.2.1 Psychological transportation

Transportation was measured using Green and Brock’s (2000) Transportation Scale adapted to suit the medium, e.g., “I was mentally involved in the narrative while watching the show”. Responses on 12 items were recorded on a scale from 1 (*Strongly Disagree*) to 7 (*Strongly Agree*), Cronbach’s α = 0.834.

##### 2.4.2.2 Parasocial interaction

The Audience-Persona Interaction Scale (APIS) was used to assess parasocial interaction (Auter and Palmgreen, 2000). Responses to the 22 items were made from 1 (*Strongly Disagree*) to 5 (*Strongly Agree*) in reference to a specific “favorite” character, e.g., “I care about what happens to FAVE”. Items were adapted to suit the medium, Cronbach’s α = 0.908.

##### 2.4.2.3 Relatedness needs satisfaction

The Assessment of Media Engagement and Satisfaction (AMES) Questionnaire was used to measure relatedness needs satisfaction (Adachi et al., 2018), e.g., “The show makes me feel a sense of attachment to the characters.” Responses on 4 items were measured from 1 (*Not at all true*) to 5 (*Very true*), Cronbach’s α = 0.952.

##### 2.4.2.4 Competence needs satisfaction

A combination of the Viewing Competence subscale (4 items) from the AMES Questionnaire (Adachi et al., 2018) and adapted items (2 items) from the Player Experience of Need Satisfaction (PENS) scale (Ryan et al., 2006) was created to measure competence needs satisfaction, e.g., “My ability to understand the plot and game was well-matched with the show’s complexity.” Responses to the six items were measured on a 1 (*Not at all true*) to 5 (*Very true*) scale, Cronbach’s α = 0.940.

##### 2.4.2.5 Comprehension

Objective comprehension of plot and game mechanics was assessed using 6 multiple-choice questions (3 each). For example, one plot comprehension question asked, “Who got new magical powers in the episode?” and displayed four character names. A sample mechanics comprehension question asked, “Based on what you observed in this video, what do you think it means when a player has advantage on a roll?” and offered four choices.

##### 2.4.2.6 Intrinsic viewing motivation

The AMES includes a 9-item subscale to measure intrinsic viewing motivation, or how interested the respondent is in continuing to watch the series or video (Adachi et al., 2018). Responses were measured on a 1 (*Not at all true*) to 5 (*Very true*) scale; example item: “Given the chance, I would continue to watch this show in my free time”; Cronbach’s α = 0.964. To obtain a behavioral measure of interest in the program outside of the lab setting, we also included a single item in which participants could provide their email address to receive a link to the original, unedited version of our stimulus.

##### 2.4.2.7 Expertise

Expertise with actual-play shows was measured using three items [e.g., *Have you played a video game that uses rules from Dungeons and Dragons* (e.g., *Baldur’s Gate*)?]. Participants were considered “experts” if they had a total score of 3 or higher on these items.

## 3 Results

Dataset and syntax are available on the project’s OSF page.

### 3.1 Descriptive statistics

Table 1 displays descriptive statistics and bivariate correlations. As expected, psychological transportation was positively associated with all measures of interest (excepting mechanics comprehension). It is particularly interesting to note the strong associations between intrinsic viewing motivation and transportation (*r* = 0.72, *p* < 0.001), parasocial interaction (*r* = 0.54, *p* < 0.001), relatedness (*r* = 0.72, *p* < 0.001), and competence need satisfaction (*r* = 0.59, *p* < 0.001).

**Table 1:** Descriptive statistics and bivariate correlations.

Variable	*M*	*SD*	1	2	3	4	5	6	7
1. Transportation	3.99	0.88	1.00						
2. Parasocial interaction	3.00	0.59	0.53***	1.00					
3. Relatedness need satisfaction	2.38	1.11	0.62***	0.56***	1.00				
4. Competence need satisfaction	3.72	0.95	0.59***	0.24**	0.43***	1.00			
5. Intrinsic viewing motivation	2.64	1.17	0.72***	0.54***	0.72***	0.59***	1.00		
6. Plot comprehension	2.32	0.71	0.31***	0.08	0.15	0.41***	0.25***	1.00	
7. Mechanics comprehension	1.62	0.90	0.08	0.03	0.03	0.11	0.08	0.17*	1.00

*p ≤ 0.05, **p < 0.01, ***p < 0.001.

Means and standard deviations for comprehension measures represent correct responses (i.e., on average, participants responded correctly to 1.62 of the mechanics comprehension items).

### 3.2 Narrative engagement

Results of independent samples *t*-tests indicated that, as hypothesized, participants in the plot-focus condition reported greater psychological transportation than did participants in the mechanics-focus condition (*t*(143.44) = 3.40, *p* < 0.001, Cohen’s *d* = 0.53) and reported greater parasocial interaction (*t*(172) = 2.71, *p* = 0.007, Cohen’s *d* = 0.41), see Table 2 for means and standard deviations.

**Table 2:** Descriptive statistics by condition.

Variable	Transportation	Parasocial interaction	Relatedness need satisfaction	Competence need satisfaction	Plot comprehension	Mechanics comprehension	Intrinsic viewing motivation
Plot-Focus Mean (SD)	4.19 (0.73)	3.11 (0.51)	2.58 (1.12)	3.82 (0.87)	2.36 (0.69)	1.68 (0.93)	2.86 (1.13)
Mechanics-Focus Mean (SD)	3.74 (0.98)	2.87 (0.64)	2.15 (1.05)	3.60 (1.03)	2.28 (0.75)	1.55 (0.87)	2.38 (1.17)

As predicted, participants in the plot-focus condition reported greater relatedness need satisfaction than those in the mechanics-focus condition, (*t*(172) = 2.60, *p* = 0.010, Cohen’s *d* = 0.40), and there was a strong, positive association between relatedness need satisfaction and parasocial interaction (*r* = 0.56, *p* < 0.001).

### 3.3 Comprehension

Contrary to predictions, those in the plot-focus condition did not comprehend more of the plot than the mechanics-focus condition (*t*(172) = 0.80, *p* = 0.426). Additionally exploratory analyses indicated that there was not a significant effect of condition upon mechanics comprehension (*t*(172) = 0.95, *p* = 0.342). Similarly, comprehension of game mechanics was not associated with feelings of competence (*r* = 0.108, *p* = 0.157). Plot comprehension, however, was positively associated with competence (*r* = 0.41, *p* < 0.001). Results of exploratory *t*-tests indicated that participants in the plot-focus condition did not report greater competence need satisfaction than those in the mechanics-focus condition (*t*(172) = 1.48, *p* = 0.140).

### 3.4 Intrinsic viewing motivation

We explored participants’ intrinsic interest in continuing to view the show using (a) the AMES Intrinsic Viewing Motivation subscale and (b) a request for receiving an emailed link of the show. Individuals in the plot-focus condition reported greater intrinsic viewing motivation than did those in the mechanics-focus condition, *t*(172) = 2.70, *p* = 0.008. However, this effect did not carry over to the behavioral measure, in which there was no significant between-group difference for requesting an emailed link to the original episode (*X*^2^ (1, *N* = 174) = 0.392, *p* = 0.531).

## 4 Discussion

The present investigation examined first-time actual-play show viewers’ experiences engaging with this emerging narrative media. Despite the complexities inherent to actual-play shows (e.g., integration of gameplay, multiple channels of communication, lack of physical narrative space), new viewers reported feeling transported into the story world and engaging in parasocial experiences with characters, and these experiences were stronger for those who focused attention on narrative components than those who focused on game mechanics. It is possible that the inherently familiar structure of narratives offers a route by which new viewers can “bypass” the complexity of the novel medium. Indeed, commonalities between traditional media and interactive storytelling have been noted as minimizing the sometimes daunting premise of new media (Roth et al., 2011). Similar processes might be at work here—although actual-play shows are not (typically) interactive, they share some common factors, such as breaks in narrative flow. This work suggests that narrative elements rather than gameplay drive engagement with actual-plays for first-time viewers.

We must note that the present findings do not indicate that focusing on mechanics when viewing these games is always undesirable. Indeed, it is likely that for many experienced viewers the gameplay itself is a core part of the viewing experience. Rather, we offer one route by which new audiences can begin to enjoy novel narrative media. Even some novice viewers, perhaps those more familiar with videogame role playing games, may become engaged with actual-play shows through the gameplay rather than the narrative. The boundary conditions of the benefits of narrative-focused audiences remain to be discovered, as do the effects of attending to game mechanics in more natural settings (i.e., when not directed to note rolls).

The present findings illustrate how overcoming barriers to emerging media can be enjoyable, and even satisfy viewers’ relatedness and competence needs. This finding is consistent with recent work highlighting how engaging with narrative entertainment could contribute to perceptions of living a more meaningful life (e.g., Oliver and Raney, 2011). Research suggests that such benefits may be associated with complex and rich entertainment use (Wirz et al., 2025) and the present studies highlight that novel, emerging media might provide these opportunities, especially when viewers are encouraged to focus on familiar narrative characteristics. It must be noted, however, that competence need satisfaction was associated only with plot comprehension, not mechanics comprehension. These findings need to be investigated further to allow for interpretation, as there are several potential explanations for this effect (e.g., third variables affecting both plot comprehension and competence, measures of comprehension limited to short multiple choice questions, etc.).

### 4.1 Limitations and future directions

The present research was restricted to a university sample. This sample was chosen because young adults are a target demographic of many actual-play shows and associated production companies (e.g., Dropout), but more work should explore narrative processes with more diverse samples, including diversity in age and ethnicity as well as diversity in media use motivations.

Diversity of actual-play shows must also be considered. The stimulus used in the present study was specific in genre (i.e., comedy), production quality (i.e., high, filmed in person, edited both by the production company and for research purposes), and performers (i.e., recognizable professional performers). The stimulus used was audiovisual, whereas many actual-play shows are produced or enjoyed only as audio-only podcasts. Future work should investigate whether results replicate in audio-only shows, and make use of stimulus sampling to improve generalizability across genre, style, production quality and company, and performers.

Additionally, experimental conditions should include a control condition given no specific instructions, or a narrative condition that includes an active task to address the multiple factors that differ between the conditions presented here. Inclusion of a second, more nuanced mechanics-focus condition could also allow future researchers to disentangle the attention effects from other factors at work in the current design (e.g., different levels of cognitive load and task demand). It is possible that distraction, more broadly, explains some or all of the effects seen here. With the inclusion of additional conditions (including a control without explicit instructions), the mechanisms underlying the effects reported in the present work can be clarified.

Future work should examine engagement processes among actual-play experts as well as novices. Experts might be better at integrating narrative and gameplay, and might be more accustomed to the collaborative and oral nature of such stories. More work is needed to explore how experiences might shift over time as expertise develops. Future work should identify best practices for measuring intrinsic motivation beyond self-report, as the behavioral measure of intrinsic interest used here may have been affected by privacy concerns.

### 4.2 Concluding thoughts

In this brief research report, we have presented a single study investigating narrative engagement with actual-play shows. This line of work is meant to highlight these shows and processes for two purposes. The first is academic in nature: Through collaboration, improvisation, and multi-layered communication, actual-play shows offer unique opportunities to better understand the processes of viewing entertainment media. The second is practical: As new media continue to develop (including actual-play), producers and artists alike benefit from a better understanding of their audience. The present results highlight the power of narrative immersion to overcome barriers inherent in emerging media, suggesting a hopeful future for storytellers looking to craft new tales by ceding control and “honoring the dice” (Lee Mulligan, 2021).

## Data Availability

The datasets presented in this study can be found in online repositories. The names of the repository/repositories and accession number(s) can be found at: https://osf.io/4px9w/overview?view_only=1047188822354f3da14221c723e4272b.

## Footnotes

1 We will use the term “viewer” to refer to narrative audience members to correspond with audiences who view audiovisual actual-play shows, the focus of this article, but please note that this work extends to multiple modalities of audiences.

2 As this investigation was particularly focused on barriers to entry and new viewer engagement, individuals with expertise in tabletop role-playing games were excluded.
